# Breeding Phenology of Birds: Mechanisms Underlying Seasonal Declines in the Risk of Nest Predation

**DOI:** 10.1371/journal.pone.0065909

**Published:** 2013-06-12

**Authors:** Kathi L. Borgmann, Courtney J. Conway, Michael L. Morrison

**Affiliations:** 1 School of Natural Resources and the Environment, University of Arizona, Tucson, Arizona, United States of America; 2 U.S. Geological Survey, Idaho Cooperative Fish and Wildlife Research Unit, College of Natural Resources, University of Idaho, Moscow, Idaho, United States of America; 3 Department of Wildlife and Fisheries Sciences, Texas A&M University, College Station, Texas, United States of America; Phillip Island Nature Parks, Australia

## Abstract

Seasonal declines in avian clutch size are well documented, but seasonal variation in other reproductive parameters has received less attention. For example, the probability of complete brood mortality typically explains much of the variation in reproductive success and often varies seasonally, but we know little about the underlying cause of that variation. This oversight is surprising given that nest predation influences many other life-history traits and varies throughout the breeding season in many songbirds. To determine the underlying causes of observed seasonal decreases in risk of nest predation, we modeled nest predation of Dusky Flycatchers (*Empidonax oberholseri*) in northern California as a function of foliage phenology, energetic demand, developmental stage, conspecific nest density, food availability for nest predators, and nest predator abundance. Seasonal variation in the risk of nest predation was not associated with seasonal changes in energetic demand, conspecific nest density, or predator abundance. Instead, seasonal variation in the risk of nest predation was associated with foliage density (early, but not late, in the breeding season) and seasonal changes in food available to nest predators. Supplemental food provided to nest predators resulted in a numerical response by nest predators, increasing the risk of nest predation at nests that were near supplemental feeders. Our results suggest that seasonal changes in foliage density and factors associated with changes in food availability for nest predators are important drivers of temporal patterns in risk of avian nest predation.

## Introduction

The reproductive period is a critical time for many organisms; individuals must resolve when, where, and how many offspring to produce. Both the timing of reproduction and the number of offspring produced per breeding attempt can have important fitness consequences and are considered primary life-history traits [1]–[4]. For example, reproductive success declines with date in a variety of organisms including many fish, insects, birds, and mammals [5]–[9]. Individuals that breed later in the breeding season typically produce smaller broods or have offspring of lower quality [1], [4], [7]. The probability of complete brood mortality can also vary with breeding date [5]–[9], but we know little about the effects of breeding date on other reproductive parameters. Despite many studies seeking to identify the mechanism(s) underlying this common pattern, the cause(s) of the seasonal decline in reproductive success are still debated [10]–[12], in part because experiments cannot manipulate timing of breeding without also affecting individual quality. More importantly, date per se cannot explain the cause of the pattern because date itself is not a selective pressure; some process associated with date must be responsible for the seasonal decline in reproductive success. Unfortunately, past studies have rarely tested more than one mechanistic hypothesis to explain the underlying cause of the seasonal decline in avian reproductive success (but see [13], [14]).

Most studies that have examined seasonal patterns in avian reproductive success have focused on seasonal declines in avian clutch size [2], [3], [11]. However, seasonal changes in avian reproductive success observed in many species are likely influenced by more than just seasonal variation in clutch size. For example, the risk of nest predation may vary seasonally but relatively few studies have examined how temporal variation in probability of nest predation contributes to seasonal declines in avian reproductive success. This oversight is surprising given that nest predation is the primary cause of nest failure in most birds [15], [16], and influences the evolution of clutch size and many other life-history traits [17]–[19]. Although breeding early is often assumed to be beneficial [due to seasonal declines in clutch size; 4,20], seasonal variation in the risk of nest predation may also affect breeding phenology. For example, if nest predation is high early in the breeding season [21], [22], the increased risk of nest predation could counteract benefits gained from a larger clutch for individuals that breed early in the season. Such a pattern would create a dilemma, especially for single-brooded passerines with limited renesting frequency. Thus, an important trade-off may exist if birds face both seasonal declines in the risk of nest predation and seasonal declines in clutch size. Such a trade-off warrants closer scrutiny because a trade-off of this nature may help explain inter- and intra-specific variation in the timing of breeding and might help explain the diversity of life-history strategies in coexisting birds.

To better understand the tradeoffs between avian breeding phenology and reproductive success, we need more information on (1) the extent to which the risk of avian nest predation changes during the breeding season, and (2) the underlying mechanism(s) responsible for any seasonal changes in nest predation. Numerous authors have reported that the risk of nest predation changes during the breeding season [22]–[25], but surprisingly few empirical studies have examined how and why nest predation changes with breeding date.

We examined these questions in Dusky Flycatchers (*Empidonax oberholseri*) nesting in montane meadows in northern California, where nests were at greater risk of predation early in the breeding season [26]. We considered six mechanistic hypotheses that may explain why the risk of nest predation declines seasonally at our study site: (1) foliage phenology, (2) energetic demand, (3) developmental stage, (4) predator search image, (5) alternative prey, and (6) predator abundance. By doing so, we hope to help clarify the constraints on breeding phenology and improve our understanding of the various life history tradeoffs that birds face when making decisions about when to breed and how many offspring to produce.

### Hypotheses

#### Foliage phenology

The foliage-phenology hypothesis relies on the same mechanism that underlies the nest-concealment hypothesis, a hypothesis that is often implicated as the cause of spatial or interspecific patterns in nest predation. This hypothesis suggests that dense foliage inhibits the transmission of auditory, visual, or olfactory cues that predators use to locate nests [15], [27]. Thus, nests surrounded by dense foliage should have a lower risk of nest predation compared to nests with less foliage. If foliage density explains the seasonal decline in nest predation on our study site, then foliage density must increase with date, such that nests initiated early in the breeding season are surrounded by less foliage compared to nests initiated later. Moreover, the foliage-phenology hypothesis explicitly predicts that seasonal changes in the risk of nest predation will be more strongly associated with seasonal changes in foliage density compared to the date only model.

#### Energetic demand

Metabolic demands of incubating females can increase by 50–90% in small-bodied songbirds when ambient temperature is 0°C relative to when ambient temperature is 15°C [28]. In montane and north-temperate ecosystems, songbirds begin breeding when night time temperatures are often <0°C (K. Borgmann, unpublished data). Length of off- and on-bouts decrease when ambient temperatures fall below physiological zero (26°C), resulting in more frequent foraging trips as females need to balance their own metabolic needs with the thermal needs of their developing embryos [29]. Alteration of incubation patterns as a result of changes in ambient temperature can subsequently increase the amount of activity around a nest site which, in turn, could attract predators to the nest site [30]–[32] and may explain why nest predation is higher early in the season. Hence, the energetic-demand hypothesis predicts that ambient temperature should explain more variation in the risk of nest predation than date alone, such that the risk of nest predation should be negatively associated with ambient temperature <26°C (the temperatures below which embryonic development is suspended; [29]).

#### Developmental stage

The developmental-stage hypothesis suggests that nest predation differs among developmental stages of the nesting cycle. Although previous studies have found that the risk of nest predation is often higher during the nestling stage due to parental activity and nestling vocalizations around the nest that attract predators [30], other studies have reported higher risk of predation during the incubation stage [33], [34]. Risk of nest predation could be higher during laying and incubation if, for example, the dominant nest predators in the system are more likely to eat eggs than nestlings [35]. If differences in the risk of nest predation among developmental stages explain the seasonal decline in the risk of nest predation in our system, then: (1) nest predation should be higher during the incubation stage than the nestling stage, and (2) risk of nest predation should not vary with date *within* each of the developmental stages of the nesting cycle.

#### Predator search image

The predator search-image hypothesis suggests that variation in nest predation is caused by predators developing search images in response to increases in nest density (i.e., density-dependent predation) [36]–[38]. Predators may become more efficient foragers as nest density increases by preferentially searching locations that previously resulted in a reward. In other words, this hypothesis posits that high nest density results in a functional response by nest predators. Intensification of a predator’s search image can increase the risk of nest predation, especially if nests are located in similar microhabitats [36], [37]. If seasonal changes in nest density explain seasonal patterns in nest predation via predator search image, then the daily risk of nest predation should be positively associated with the number of active nests. For this hypothesis to explain the seasonal decline in nest predation observed in our system, nest density must peak early in the breeding season.

#### Alternative prey

The alternative-prey hypothesis suggests that predators alter their search tactics and switch to foraging for nest contents (alternative prey) when the abundance of their primary prey is low, as suggested by optimal foraging theory [39], [40]. Cold temperatures early in the season may limit the availability of insect [41] and cone crops [42], [43] that are a major food source for many common nest predators of North American songbirds [e.g., jays (*Cyanocitta* spp.), chipmunks (*Tamias* spp.), and squirrels (*Tamiasciurus* spp., *Sciurus* spp.)]. Thus, nest predators may be forced to search for nest contents early in the season when their primary food resources are less abundant. If the alternative-prey hypothesis explains high nest predation early in the breeding season, then supplementing nest predators with food early in the breeding season (when their primary food is presumably less abundant) should cause predators to reduce the amount of time spent searching for nests, subsequently reducing the risk of nest predation. In other words, providing predators with additional food early in the breeding season should result in predator satiation via a functional response. However, providing supplemental food could also alter the spatial distribution of nest predators via a numerical response [44], which could increase the risk of nest predation for nests located near the supplemental feeders [45], [46]. Thus, if supplemental food causes nest predators to alter their spatial distribution, then the risk of nest predation should increase near the supplemental feeders as predators concentrate their foraging in these prey-rich patches (a key feature of the enemy-free space hypothesis; [46]).

#### Predator abundance

The predator-abundance hypothesis suggests that seasonal variation in nest predation is caused by seasonal variation in predator density. If a change in predator abundance explains the seasonal decline in nest predation in our system, then seasonal changes in predator abundance should mimic seasonal changes in the risk of nest predation such that predator abundance should be positively correlated with risk of nest predation across study sites.

## Materials and Methods

### Ethics Statement

Permission to conduct field studies was granted by the United States Forest Service Lake Tahoe Basin Management Unit. Field studies did not involve endangered or protected species. No animals were harmed in this study and no animals were captured or handled. Surveys of birds and small mammals were conducted passively. This project was approved by The University of Arizona Institutional Animal Care and Use Committee (IACUC #06–049).

### Study Area

We examined seasonal patterns of predation on Dusky Flycatcher nests from 2006 to 2008 in montane meadows surrounding Lake Tahoe, California (38° 56′ N 119° 59′ W). We also tested the predictions listed above to provide insight into which hypotheses best explained the seasonal patterns that we observed. We monitored nests within five 10–20 ha montane meadows that ranged in elevation from 2000 to 2390 m. Sites were dominated by willows (primarily *Salix lemmonii* and *S. geyeriana*), sedges (C*arex* spp.), rushes (*Juncus* spp.), and numerous herbaceous flowering plants. Mixed-conifer forest with lodgepole pine (*Pinus contorta*), white fir (*Abies concolor*), and Jeffrey pine (*P. jeffreyi*) surrounded each meadow. Patches of quaking aspen (*Populus tremuloides*) also occurred along meadow edges.

### Nest Searching and Monitoring

We located 185 Dusky Flycatcher nests primarily by following females carrying nesting material or food to their nests. We discovered 64% of the nests prior to clutch completion. We monitored nests every two to four days until the offspring fledged from the nest or the nest was depredated [47]. We considered a nest successful if we observed parents feeding at least one offspring outside of the nest or if we observed fecal matter on the rim of the nest cup. Nests in which eggs or nestlings disappeared prior to their expected fledging date were considered depredated. Because our goal was to assess the risk of nest predation, we considered nests that fledged only Brown-headed Cowbirds (*Molothrus ater*) as successful (*n* = 2). We did not include nests where parental activity at the nest ceased but eggs remained intact (i.e., abandoned nests; *n* = 4) or nests with uncertain nest fates (*n* = 1) in our analysis.

### Foliage Phenology

We tested the foliage phenology hypothesis by measuring changes in foliage density at nest sites throughout the breeding season. We recorded foliage density measurements (1) at the time of nest failure or success, (2) at the expected fledge date if the nest failed, and (3) each week at nest sites that were active during one of the two previous summers (2006 or 2007). We collected weekly foliage measurements at prior-year nest sites to avoid the harassment of adults that would have occurred with weekly foliage measurements at active nests. We used two approaches to measure average foliage density at Dusky Flycatcher nest sites: (1) estimated visually the percent of the nest site obscured by vegetation, and (2) calculated the percent of the nest site obscured by vegetation from digital photographs of the nest. Estimating the percent of a Dusky Flycatcher nest site obscured by vegetation is often problematic because Dusky Flycatchers frequently reuse nesting material from failed nests (i.e., nests often disappear soon after failure, eliminating the point of reference). Hence, once a nest was no longer active we secured a 12.7 cm orange styrofoam ball at the location of the nest and left the styrofoam ball in place for the remainder of the breeding season. An observer stood 1 m from the orange ball and visually estimated the percent of the ball obscured by vegetation and also took a digital photograph (Kodak EasyShare C813 camera) of the ball while standing at the same location. The observer obtained visual estimates and took photographs at each nest site at two height intervals (0.5 m and 1 m) from each of four cardinal directions (North, South, East, and West) around the nest. We used MATLAB (Version 7.8 R2009a) image processing to calculate the percentage of orange pixels in each image and to determine the average percentage of foliage obscuring the nest. Image processing in MATLAB required that we crop each image to include only the orange ball and train the program to recognize orange-colored pixels through an iterative process on a group of sample images to create a color palate. Once we input the color palate into MATLAB, we calculated the number of orange-colored pixels in each image as a measure of foliage density.

We used digital photographs and visual estimates to quantify foliage density at Dusky Flycatcher nests that were active only in 2008 and to record weekly foliage density measurements at nests active in previous years. In the previous two years (2006 and 2007), we used a density board to measure foliage density at active nests. We placed a 0.25m^2^ density board at the center of each nest and an observer counted the number of white squares visible while looking at the density board at two height intervals (0.5 m and 1.0 m) while standing 1 m from the nest. Observers recorded the two density board readings at each of four cardinal directions around the nest. Although density board measurements are commonly used to estimate foliage density around bird nests [48], [49], they had low repeatability across time when measuring foliage density weekly due, in part, to the challenges of placing the density board in the same location on subsequent visits (K. Borgmann, unpublished data). Hence, we used estimates of foliage density obtained from the digital photographs in 2008 to assess the effect of foliage phenology on daily nest survival of Dusky Flycatchers for all years of the study (2006–2008). Although foliage density at a specific nest site can change annually, we believe that the annual variation was small relative to the extent of seasonal change in foliage density (seasonal foliage maturation) at our sites.

### Energetic Demand

We evaluated the potential effect of seasonal changes in energetic demand for incubating females by recording ambient temperature at our study sites. Ambient temperature in cold, montane environments is thought to affect energetic demand and incubation behavior [29]. We calculated the proportion of the day in which the temperature was below 26°C because length of off- and on-bouts decrease when ambient temperatures fall below physiological zero (26°C). In 2006, we used hourly temperature data collected at the Lake Tahoe Regional Airport (National Climate Data Center) near our study sites (distance from our study sites ranged from 11 to 33 km). In 2007 and 2008, we measured temperature with Thermochron i-buttons (DS1921k Maxim Integrated Products, Sunnyvale, CA) placed at the center of each study site.

### Predator Search Image

We used the Horvitz-Thompson estimator [23], [50] to estimate the number of active nests for each day of the breeding season. The Horvitz-Thompson estimator allowed us to appropriately account for the nests that we failed to locate ['adjusted nests' in 23].

### Alternative Prey

We provided potential nest predators with dried corn, black oil sunflower seeds, and whole in-shell peanuts to test the alternative-prey hypothesis at three of our study sites in 2007 and 2008. The most common potential nest predators at our sites included Steller’s Jays (*C. stelleri*), Clark’s Nutcrackers (*Nucifraga columbiana*), chipmunks, and squirrels [51], all of which will readily consume corn and seed. At each of our food-supplemented sites, we placed supplemental food (726 g of food per feeder) in ten bird feeders 40 m apart along the ecotone between meadow and forest. We also scattered additional food on the ground while walking between feeders to further decentralize the distribution of supplemental food and to provide supplemental food for predators that may have had difficulty accessing the feeders (e.g., *Peromyscus maniculatus*). Feeders were filled regularly (typically once or twice every five days) beginning 19 May in 2007 and 21 May in 2008. We provided supplemental food for approximately one month, coinciding with the early portion of the breeding season, which is when this hypothesis assumes that the primary food of nest predators was less abundant (19 May–23 June 2007 and 21 May–27 June 2008). We opportunistically recorded predators observed at feeders while monitoring and locating nests. Because we were concerned that the supplemental food experiment might concentrate nest predators near feeders, we also conducted point-count surveys (described below) for nest predators before and after food supplementation at each site.

### Predator Abundance

We estimated abundance of avian and small mammal nest predators at our sites by conducting point-count surveys to test the predator-abundance hypothesis. Point-count surveys are commonly used to assess the abundance of vocal nest predators [52], [53] and the most common potential nest predators at our sites included Steller’s Jays, Clark’s Nutcrackers, chipmunks, and squirrels [51], all species which are easily detected by sight and sound during daylight hours. We established three to four point-count stations at each of our five study sites. The number of point-count stations varied among sites due to variation in the size of our study sites. We placed point-count stations 250 m apart along the ecotone between meadow and forest within our study sites. We surveyed each site once per week in 2007. Observers recorded all potential nest predators seen or heard within 50 m of the point-count station during a 10-minute survey at each point. We averaged the number of nest predators detected across all survey points within a site for each week of the breeding season. We separated predator abundance into avian and mammalian nest predators because foraging strategies are often assumed to differ between birds (visual) and mammals (olfactory and visual) [54], [55].

### Modeling Nest Predation

We used the logistic exposure method [56], [57] to model nest survival and calculated the daily probability of nest predation (1-nest survival). The logistic exposure method allows nest predation to be modeled based on a suite of covariates that can be continuous, categorical, or time-varying. The logistic exposure method uses the intervals between nest checks as the sampling unit to interpolate daily survival estimates. We modeled nest predation with PROC GENMOD and the logit link function in SAS [57], [58]. We first examined how the risk of nest predation changed during the breeding season. We included all Dusky Flycatcher nests that were not part of an experimental treatment (i.e., we did not include nests on food-supplemented sites) to assess the overall pattern of seasonal changes in nest predation [26]. We used an information-theoretic approach to evaluate four models [59] to examine if and how nest predation changes during the breeding season: (1) constant survival (i.e., risk of nest predation was constant throughout the breeding season), (2) linear date term, (3) quadratic date term, and (4) cubic date term. We included quadratic and cubic date terms because several studies have reported non-linear relationships between nest survival and date [60]–[63]. We did not include year as a nuisance variable in our models because preliminary examination of the data indicated that nest predation was similar among the three years (K. L. Borgmann, unpublished data). We regarded models with ΔAIC_c_ values <4.0 and evidence ratios <5.0 as equally plausible [59], [64]. We used the effective sample size to calculate AIC_c_ [65].

Next, we compared the best model identified in the process described above (1-Survival = f(Date+Date^2^)) with a suite of mechanistic models (three for each of the hypotheses plus the null model; Table 1). This two-step model selection process allowed us to evaluate whether any of the models that included explicit mechanistic factors (i.e., foliage phenology, predator abundance, etc.) performed better than our best “date only” model and, hence, could explain why nest predation varied with date. We did not include models with multiple mechanisms (i.e., with energetic demand and predator search image) or interaction terms among the mechanistic factors because: (1) we designed our study from the outset to test among the six mechanistic hypotheses to determine which factor best explained the observed seasonal decline in nest predation, (2) the number of possible models involving additive and interactive effects among mechanistic factors is enormous, and (3) our analytical approach modeled the risk of failure on a daily basis and the number of samples (nests) for any given day was not always high even with our large sample of nests to adequately test for interactions between mechanistic causes.

**Table 1:** Explanation of the models used to examine proposed mechanisms that could affect seasonal changes in the risk of nest predation.

Model	Explanation
1-Survival = f (Date+Date^2^)	Nest predation varies non-linearly with date
1-Survival = f (Mechanistic Factor)	The proposed mechanistic factor is responsible for the observed seasonal decline in nest predation independent of date
1-Survival = f (Mechanistic Factor+Date+Date^2^)	Nest predation varies non-linearly with date and the proposed mechanistic factor explains additional variation in nest predation
1-Survival = f (Mechanistic Factor+Date+Date^2^+ Mechanistic Factor *Date+Mechanistic Factor *Date^2^)	The non-linear relationship between nest predation and date is affected by the proposed mechanistic factor.

We generated model-based estimates of the risk of nest predation after we fit models with the logistic exposure method to determine the effect of individual covariates. We used model-averaged parameter estimates and 95% confidence intervals in cases of model selection uncertainty (i.e., when no one model was clearly supported) [57], [59]. In cases where one model best supported the data, we reported estimates of nest predation based on the single-best model. We produced estimates of nest predation while holding date at representative values throughout the breeding season to visualize how individual time-varying covariates affected nest predation [57]. For example, we calculated estimates of daily risk of nest predation for values of foliage density at seven representative dates during the breeding season that corresponded to the range of nest initiation dates that we observed during our study.

We conducted separate model selection procedures for the food-availability and predator-abundance hypotheses due to differences in the subsets of nests (and hence the sample sizes) available to test these two hypotheses following the procedures outlined above.

## Results

**Figure 1:**
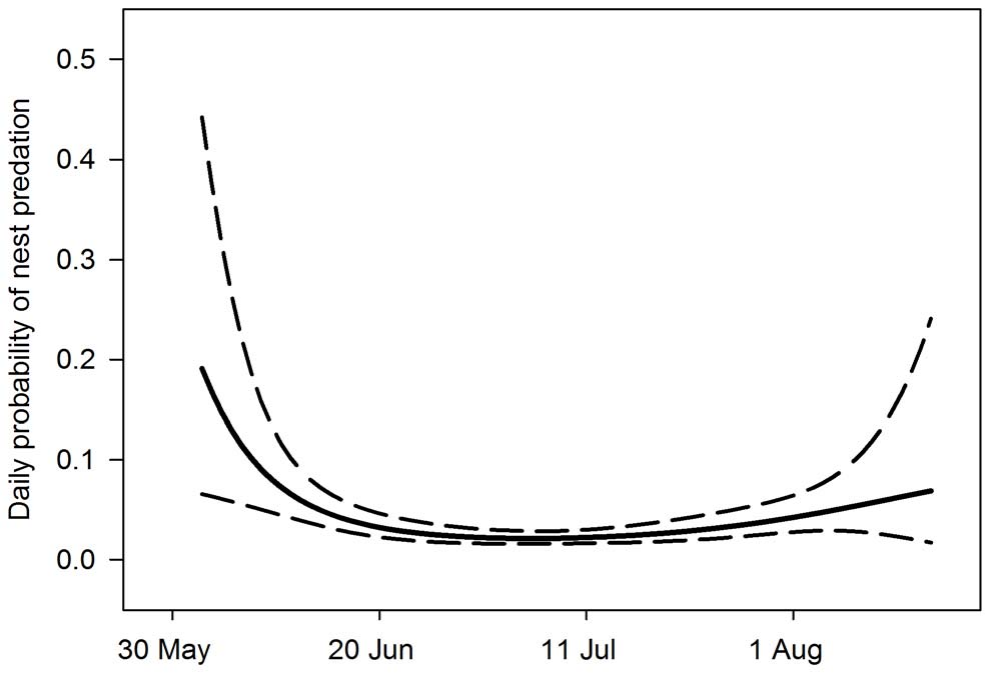
Seasonal variation in the daily probability of nest predation. Estimates of the daily probability of nest predation in Dusky Flycatchers (solid line) with 95% upper and lower confidence limits (dashed lines) generated from the best-supported model in step one of our modeling approach (Nest predation = Date+Date^2^; Table 1).

We monitored 185 Dusky Flycatcher nests from 2006 to 2008. Predation accounted for 94% of nest failures. Risk of nest predation followed a curvilinear pattern in which daily nest predation was high early in the breeding season, decreased sharply, and then remained relatively constant thereafter (Fig. 1; β with 95% CL: Date = 0.150 [0.080, 0.221], Date^2^ = −0.001 [−0.002, −0.001]). The top two models included cubic and quadratic effects of date, while linear effects of date and the constant survival model had ΔAIC_c_ >10.00 (Table 2). Because the cubic term added little to overall model fit (Likelihood ratio test χ^2^ = 1.6, *P* = 0.209), we did not consider the cubic term in subsequent models. We assessed goodness-of-fit test based on a global model from the candidate set (Table 1). The Hosmer and Lemeshow goodness-of-fit test [66] indicated that the global model (linear, quadratic, and cubic date variables and covariates) fit the data well (*P* = 0.867). We assessed overdispersion by examining the ratio between χ^2^ and model degrees of freedom from the global model; our results indicated little overdispersion (ĉ <1.00).

**Table 2:** Model selection results examining the potential relationship between the risk of nest predation and date during the breeding season for Dusky Flycatchers (*n* = 167) from 2006 to 2008, Lake Tahoe, California.

Model	−2 log *L*	K	AIC_c_	ΔAIC_c_	*w_i_*
Date+Date^2^+ Date^3^	−292.37	4	592.75	0.00	0.64
Date+Date^2^	−293.95	3	593.91	1.16	0.36
Constant	−301.38	1	604.76	12.01	0.00
Date	−301.23	2	606.46	13.71	0.00

Effective sample size used to calculate AIC_c = _2882.

### Foliage Phenology

**Figure 2:**
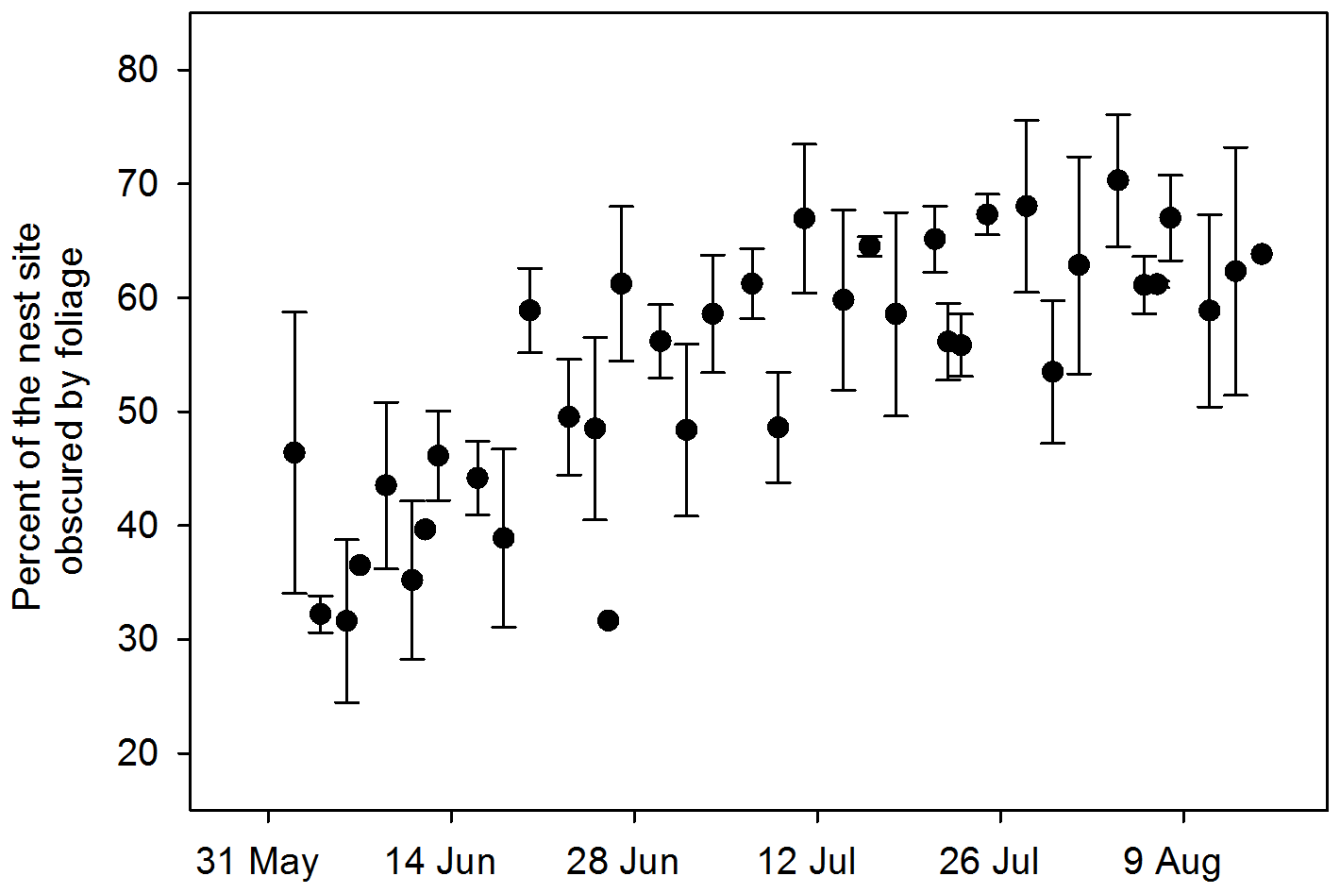
Seasonal variation in foliage density at nest sites. Percent of the nest site obscured by foliage density within 1-m radius surrounding Dusky Flycatcher nests increased with date in a non-linear fashion (Mean percent foliage density for nests on the same day ± SE). Measures of foliage density represent average nest concealment measurements for a collection of nests measured on the same day.

**Figure 3:**
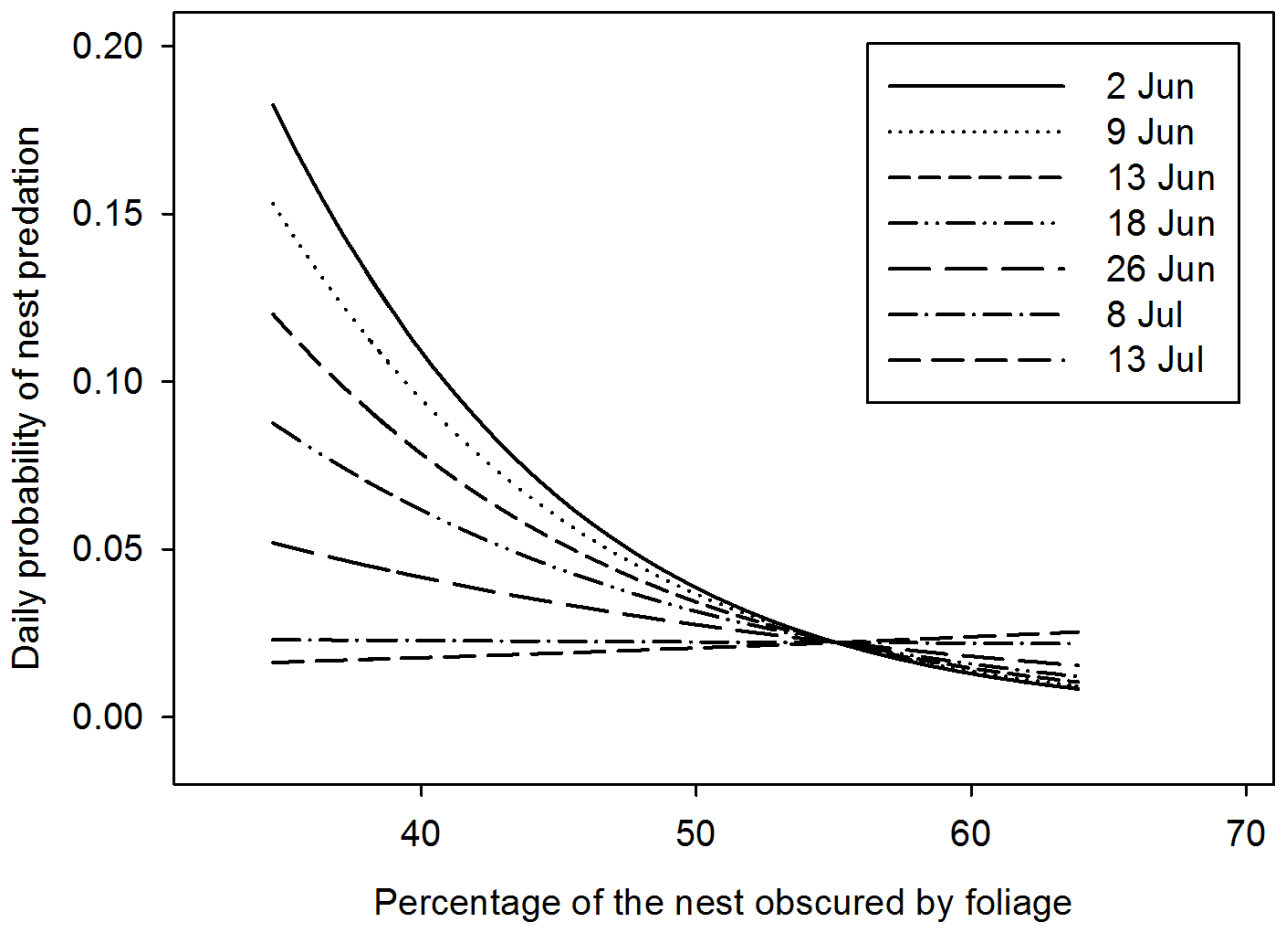
Daily probability of nest predation in relation to foliage density. Estimates of daily probability of nest predation for Dusky Flycatchers as a function of the percentage of the nest obscured by foliage generated from the best-supported model in step 2 of our modeling approach (Nest predation = Foliage density+Date+Foliage Density*Date; Table 2). The effect of foliage density on daily nest predation was assessed at seven representative dates throughout the breeding season. Daily nest predation was negatively associated with foliage density early in the breeding season (2–18 June), but not late in the breeding season (after foliage density had matured on ∼10 July; see Fig. 2).

Foliage density measured at nest sites changed with date (*F*_2,142_ = 37.43, *P*<0.001), but the pattern was not linear. The amount of foliage present at nest sites increased by 17% from the beginning of the breeding season until 1 July when foliage density reached maturity and remained relatively constant throughout the remainder of the breeding season (Fig. 2). These seasonal increases in foliage density affected the risk of nest predation for Dusky Flycatchers (Table 3). The model that included an interaction between foliage density and the quadratic date term was among the best-supported model (Table 3; β with 95% CL: Foliage*Date = −0.010 [−0.018, −0.002]; Foliage*Date^2^ = 0.000 [−0.000, −0.000]; Foliage = 0.314 [0.107, 0.521] Date^2^ = −0.005 [−0.009, 0.000]). Models with the foliage term and the interaction with the quadratic date term performed better than models with either the quadratic date term or foliage term alone suggesting that the effects of foliage density on risk of nest predation changes as the season progresses. The risk of nest predation was negatively associated with foliage density early in the breeding season, but not later in the breeding season (Fig. 3). Nests initiated early in the season had a low risk of nest predation if nests were surrounded by dense foliage, but a high risk of nest predation if the nests were not surrounded by dense foliage (Fig. 3).

**Table 3:** Model selection results examining the effect of foliage phenology (Foliage), minimum energetic demand (Temp), developmental stage (Stage), and conspecific nest density (Density) on the risk of nest predation (1-Survival) for Dusky Flycatchers (*n = *167) from 2006 to 2008, Lake Tahoe, California.

Model	log *L*	K	AIC_c_	ΔAIC_c_	*w_i_*
Stage+Date+Date^2^+Stage*Date+Stage*Date^2^	−287.40	6	586.83	0.00	0.65
Foliage+Date+Date^2^+Foliage*Date+ Foliage*Date^2^	−288.88	6	589.79	2.96	0.15
Stage+Date+ Date^2^	−290.90	4	589.81	2.99	0.15
Date+Date^2^	−293.95	3	593.91	7.08	0.02
Density+Date+Date^2^	293.10	4	594.22	7.39	0.03
Temp+Date+Date^2^	293.91	4	595.83	9.00	0.01
Foliage+Date+Date^2^	293.94	4	595.89	9.06	0.01
Density+Date+Date^2^+Density*Date+Density*Date^2^	−293.03	6	598.08	11.25	0.00
Temp+Date+Date^2^+Temp*Date+Temp*Date^2^	293.73	6	599.49	12.66	0.00
Stage	−297.80	2	599.61	12.78	0.00
Density	298.71	2	601.43	14.60	0.00
Constant	301.38	1	604.76	17.93	0.00
Foliage	300.67	2	605.34	18.51	0.00
Temp	300.85	2	605.70	18.87	0.00

Effective sample size used to calculate AIC_c_ = 2882.

### Energetic Demand

The proportion of each day in which temperature was below 26°C decreased with date until the middle of the breeding season (7 July), and then began to increase with date thereafter (*F*_2,106_ = 56.41, *P*<0.001). Changes in the proportion of each day in which ambient temperature was below 26°C had little effect on seasonal changes in the risk of nest predation; models containing temperature ranked well below the constant survival model (Table 3; β with 95% CL: Temp = 0.030 [−0.187, 0.247]; Date^2^ = −0. 0028 [−0.009, 0.004]; Temp*Date = −0.002 [−0.010, 0.007]; Temp*Date^2^ = 0.000 [−0.000, 0.000]).

### Developmental Stage

Developmental stage and the interaction between developmental stage and date were included in the best-supported models (Table 3; β with 95% CL: Stage = −8.93 [−18.832, 0.972]; Stage*Day = 0.302 [−0.010, 0.615]; Stage*Day^2^ = −0.003 [−0.005, −0.000]), suggesting that risk of nest predation did differ between developmental stages. However, the pattern was opposite that predicted by the developmental-stage hypothesis; nest predation was slightly higher during the nestling stage compared to the incubation stage. Moreover, the 95% CLs for the two parameter estimates overlapped (Incubation = 3.22 [2.95, 3.49], Nestling = 3.81 [3.46, 4.15]).

### Predator Search Image

The number of nests active per day peaked on 23 June. Seasonal changes in nest density did not correspond with the observed seasonal pattern in the risk of nest predation (Table 3). Models that included nest density all had little support. Indeed, the pattern was in the opposite direction predicted by the foraging-efficiency hypothesis; risk of nest predation was highest when nest density was lowest.

### Alternative Prey

**Figure 4:**
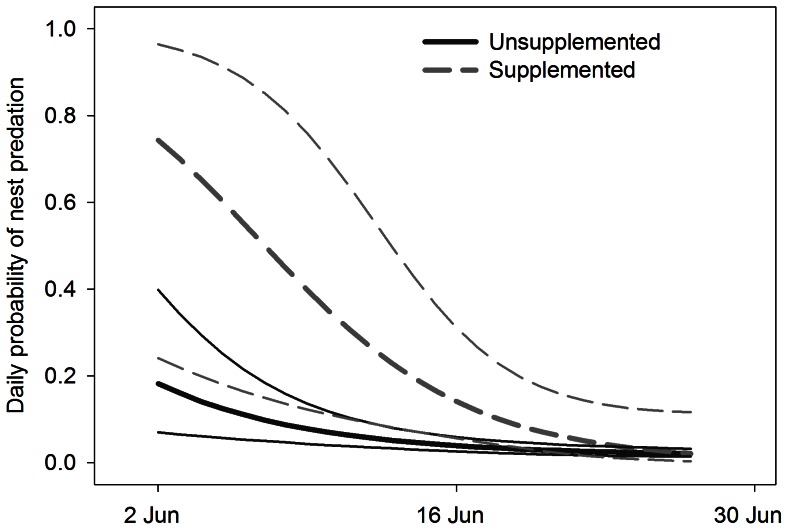
Daily probability of nest predation at supplemented and unsupplemented nest sites. Estimates of daily probability of nest predation for Dusky Flycatchers (heavy lines) with 95% upper and lower confidence limits (fine lines) generated from the best-supported model from our supplemental food experiment (Nest predation  =  Food+Date+Date^2^+Food*Date^2^; Table 4). Daily nest predation was higher in areas near feeders (dashed line) compared to areas further from feeders (solid line), but the effect dissipated later in the season.

**Figure 5:**
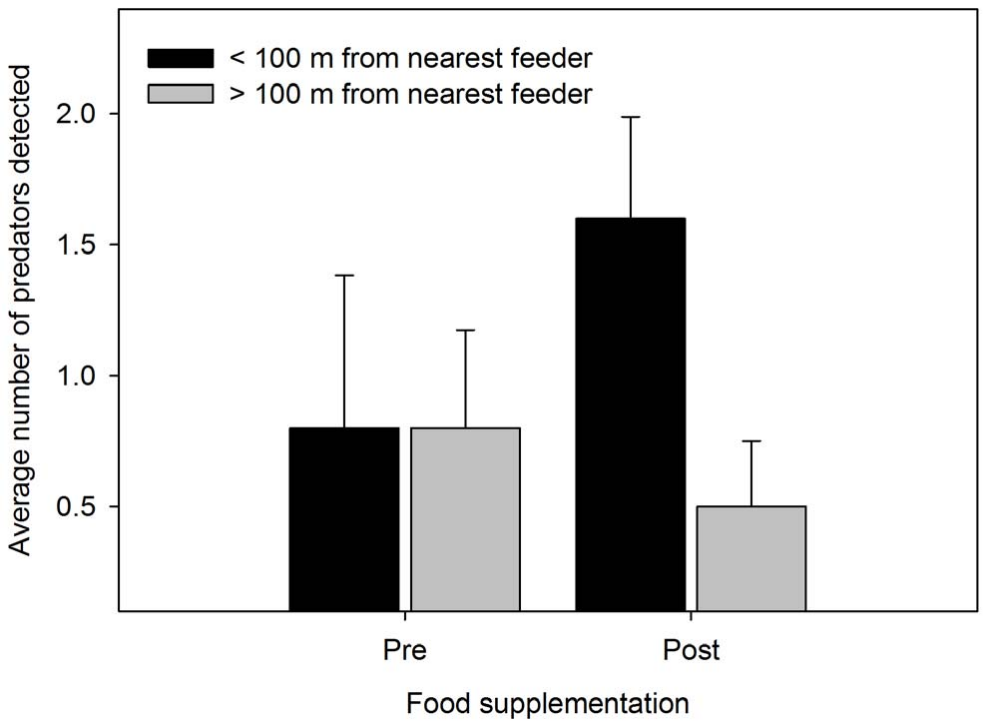
Average number of nest predators detected near and far from feeders pre- and post-supplementation. Average number (±SE) of potential avian and mammalian nest predators detected within 50 m of point-count stations <100 m from feeders (black bars) and >100 m from feeders (grey bars) for the periods both pre- and post-food supplementation. The average number of potential nest predators detected was similar prior to food supplementation at point count stations <100 m and >100 m from feeders. After food supplementation began, the average number of potential nest predators detected post-food supplementation increased at point-count stations located <100 m from feeders (black bars) but decreased at point-count stations >100 m from feeders (gray bars).

We observed Steller’s Jays, Douglas squirrels (*Tamiasciurus douglasii*), and chipmunks consuming food at the feeders. An average of 31 kg of supplemental food was consumed every week in each year of the experiment. The interaction between food supplementation and a quadratic date effect was among the best-supported models (Table 4). However, supplemental food increased rather than decreased risk of nest predation: opposite the pattern predicted by the alternative-prey hypothesis (Fig. 4). The risk of nest predation at Dusky Flycatcher nests <50 m from a feeder was higher than at nests >50 m from a feeder (Fig. 4). Prior to supplemental feeding, predator abundance was equal at point-count stations <100 m and >100 m from feeders (Fig. 5; Pre <100 = 0.8±0.58; Pre >100 m = 0.8±0.37). In contrast, predator abundance at point-count stations located within 100 m of a feeder doubled following food supplementation (Fig. 5; Post <100 m = 1.6±0.39; Post >100 m = 0.5±0.25). Hence, food supplementation created a numerical response rather than a functional response.

**Table 4:** Model selection results examining the effect of providing supplemental food for nest predators on the risk of nest predation for Dusky Flycatchers (*n = *76) from 2007 to 2008, Lake Tahoe, California.

Model	log *L*	K	AIC_c_	ΔAIC_c_	*w_i_*
Food+Date+Date^2^	−142.62	4	293.25	0.00	0.73
Food+Date+Date^2^+Food*Date+Food*Date^2^	−142.07	6	296.20	2.95	0.23
Date+Date^2^	−145.61	3	297.24	3.99	0.14
Food	−150.96	2	305.92	12.67	0.00
Constant	−153.97	1	309.94	16.69	0.00

Effective sample size used to calculate AIC_c_ = 1446.

### Predator Abundance

Potential nest predators detected during point-count surveys included Steller’s Jays, Clarks Nutcrackers, Common Ravens (*Corvus corax*), Northern Goshawks (*Accipiter gentilis*), Red-tailed Hawks (*Buteo jamaicensis*), chipmunks, and Douglas squirrels. Abundance of potential avian and mammalian nest predators changed during the breeding season (Avian, *F*_1,42_ = 13.96, *P*<0.001; Mammalian, *F*_1,42_ = 27.44, *P*<0.001). Potential avian nest predators decreased with date, whereas potential mammalian nest predators increased with date. Seasonal changes in the abundance of potential avian and mammalian nest predators were associated with the seasonal changes in risk of nest predation for Dusky Flycatchers (Table 5). Models incorporating the main effect and interaction terms of potential avian and mammalian predator abundance and a quadratic date term were among the best-supported models (Table 4).

**Table 5:** Model selection results from models examining the effects of potential avian and mammalian nest predator abundance on the risk of nest predation for Dusky Flycatchers (*n* = 83) in 2007, Lake Tahoe, California.

Model	log *L*	K	AIC_c_	ΔAIC_c_	*w_i_*
Avian+Date+Date^2^	−135.37	4	278.78	0.00	0.49
Mammal+Date+Date^2^	−135.81	4	279.66	0.87	0.32
Avian+Date+Date^2^+Avian*Date+Avian* Date^2^	−135.03	6	282.14	3.36	0.09
Mammal+Date+Date^2^+Mammal*Date+ Mammal*Date^2^	−135.12	6	281.31	3.53	0.08
Avian+Mammal+ Date+Date^2^	−139.72	4	287.48	8.69	0.00
Date+Date^2^	−140.95	3	287.92	9.14	0.00
Mammal	−142.71	2	289.43	10.65	0.00
Avian	−142.92	2	289.85	11.07	0.00
Constant	−148.78	1	299.56	20.78	0.00

Effective sample sizes used to calculate AIC_c_ = 1200.

## Discussion

Seasonal variation in probability of nest predation is not uncommon in bird populations, but the nature of the pattern appears to vary among species and ecosystems. Some studies have reported seasonal increases in risk of nest predation [24], [25], [67], [68], while others have reported seasonal decreases in the risk of nest predation [21], [69], [70]. Despite spatial variation in directionality of the relationship between breeding date and risk of nest predation, the same mechanism may still be responsible. Previous studies have suggested that the seasonal decline in the risk of nest predation was caused by seasonal changes in foliage density [71], [72] or changes in activity or behavior of predators [23], [24], [73], [74]. Our results suggest that both seasonal changes in foliage density and predator food availability can affect seasonal patterns in the risk of nest predation. While our findings are specific to the system we studied, availability of alternative prey may increase seasonally in some systems and decrease seasonally in others, potentially explaining both seasonal increases and seasonal decreases in the risk of nest predation in different systems. A comparative analysis would be particularly instructive to determine whether the same mechanism(s) is potentially responsible for variation in seasonal patterns in the risk of nest predation across systems.

While poor nest-site selection by early-arriving birds [31], [75] could contribute to the seasonal pattern in risk of nest predation that we observed in our system, we do not believe poor nest-site selection is responsible for the seasonal decrease in risk of nest predation for three reasons. First, nest-site quality is not a true alternative to the hypotheses we considered because nest-site selection does not elucidate the exact mechanism responsible for the pattern. For example, a nest site can be of ‘poor quality’ (and hence get depredated quickly) for numerous reasons: the nest site may have less vegetative cover than other sites and hence is more susceptible to predation (the mechanism in our foliage-phenology hypothesis), or more predators may be in the area (the mechanism in our predator-abundance hypothesis). Hence, the nest-site quality hypothesis implicitly assumes that vegetative cover or predator abundance or food availability (or some other process) differs between early and late nesting attempts. Second, although higher risk of nest predation early in the breeding season may be due to poor nest-site selection or poor-quality parents (Martin et al. 2000), more-experienced individuals typically begin breeding earlier, not later, in most avian communities [76], [77]. Hence, the proportion of lower-quality individuals (which likely breed in lower-quality sites) should increase as the season progresses which would create the opposite pattern than what we observed. Finally, the risk of nest predation was lower during the incubation stage compared to the nestling stage in our system, opposite of what one would predict if the seasonal pattern in predation risk that we observed was caused by nests in poor-quality sites getting depredated quickly (i.e., during the egg-laying and incubation stages).

### Foliage Phenology

We found that the risk of nest predation decreased as foliage density increased during the breeding season. Past studies have implied that foliage phenology contributed to seasonal variation in the risk of nest predation, but did not measure changes in foliage phenology [23], [25], [78], [79]. While foliage phenology appears to affect the risk of nest predation early in the breeding season in our system, we lack information regarding the precise mechanism by which the foraging ability of nest predators changes with foliage phenology. For example, visually oriented predators may be more likely to depredate nests early in the season because nests are more visible.

### Developmental Stage

Although the developmental stage was included in the best supported models, the direction of the pattern was opposite of that predicted by the developmental-stage hypothesis. If differences in vulnerability associated with developmental stage were causing the seasonal declines in the risk of nest predation that we observed, then the risk of nest predation should have been higher during the incubation stage compared to the nestling stage. Risk of nest predation, however, was higher during the nestling stage in Dusky Flycatchers. Some previous studies have reported higher nest predation during the nestling stage, presumably due to the increased activity and noise of nestlings [31]. However, stage-specific differences in the risk of nest predation have not been consistent across studies; some report a higher risk of predation during incubation while others report a higher risk of predation during the nestling stage [24]. Nonetheless, differences in nest predation among developmental stages observed in our system cannot explain the seasonal pattern in risk of nest predation.

### Alternative Prey

We observed Steller’s Jays, chipmunks, and Douglas squirrels consuming food at the feeders and we documented one instance each of a Steller’s Jay, weasel (*Mustela* spp.), and Brown-headed Cowbird depredating nests. Chipmunks, deer mice, Clark’s Nutcrackers, and Douglas squirrels have also been documented depredating nests of songbirds in the Lake Tahoe area [51] and were present on our study sites. Thus, our supplemental food was likely consumed by species responsible for at least some (and likely most) of the nest predation events at our study sites. However, we did not find support for the alternative-prey hypothesis. Nest predation increased (rather than decreased) at nests nearest to supplemental feeders. The increase in nest predation in response to our supplemental food experiment suggests that supplemental food caused a numerical, rather than a functional, response by nest predators. Predator abundance doubled near feeders after food supplementation began, but did not change in areas further from feeders, suggesting that supplemental food drew local predators into the area (at least temporarily) and subsequently increased the risk of nest predation. Spatial and temporal changes in predator abundance or foraging behavior that we observed in response to supplemental food are consistent with the enemy-free space hypothesis [45], [46]. The enemy-free space hypothesis is a facet of optimal foraging theory and assumes that a predator will exploit food-rich patches because these patches are more profitable [45], [46]. The encounter rate with nests will then increase within food-rich patches, as predators forage for other prey items in the food-rich patches, but will decrease outside of food-rich patches (i.e., in food-poor patches) because a predator spends less time foraging in these low-quality patches [46]. Hence, supplemental food redistributed local nest predators, but did not satiate them to the point where probability of nest predation declined. Our supplemental food experiment may not have accurately mimicked the typical spatial or temporal pattern of higher food abundance in the natural system. This caveat, however, is true for virtually all supplemental food experiments (positive effects imply food is important, but lack of a response to supplemental food is more difficult to interpret). We did find an effect of supplemental food at our study sites (albeit a numerical rather than functional response) suggesting that food availability (at least at a small scale) affects nest predator behavior and, subsequently, the risk of nest predation in our system.

### Predator Abundance

We found equivocal support for the predator-abundance hypothesis. Similarly, some past studies that have tested the predator-abundance hypothesis have suggested that predator abundance was positively associated with probability of nest predation [80]–[83] while others have suggested that predator abundance was negatively associated with nest predation [84], [85]. Potential avian nest predators were slightly more abundant early in the breeding season, but the Akaike weights were relatively small (<0.45). In our system, abundance of avian nest predators may have decreased during the breeding season due to altitudinal migration or dispersal. Clark’s Nutcrackers often migrate altitudinally in response to seasonal availability of their primary food resources or because of inclement weather at higher elevations [86], [87]. Indeed, we detected a greater number of Clark’s Nutcrackers early in the breeding season (K. L. Borgmann, unpublished data). Abundance of avian nest predators may also decrease late in the breeding season as individuals begin to disperse after their breeding season. Steller’s Jays initiate breeding prior to Dusky Flycatchers and may therefore begin dispersing while Dusky Flycatchers are still incubating and brooding young, leaving late nesting attempts less vulnerable to predation by Steller’s Jays.

The seasonal changes in predator abundance that we observed could also be an artifact of seasonal declines in detection probability of potential nest predators [88] because increased foliage density late in the breeding season could reduce detection probability of nest predators [89]. Although we did not account for detection probability explicitly during our surveys, we restricted our analysis to include only detections within 50 m of point-count stations. Moreover, the relative frequency of visual and auditory detections did not change with date (Mean date of aural detections = 40.6±2.3 [SE]; mean date of visual detections = 34.1±4.8 [SE]), suggesting that detection probability of potential nest predators likely did not change substantially with date. However, the evidence supporting the predator-abundance hypothesis is ambiguous. A more rigorous test of the predator-abundance hypothesis would involve examining the number of nests depredated by specific species or manipulating the predator abundance.

### Conclusion

Although migratory birds in temperate regions that breed early are often thought to gain an advantage because they typically lay larger clutches and their offspring have more time to develop and mature prior to migration, early-nesting birds also may experience a higher risk of nest predation (as in our system). Resolution of this trade-off may affect optimal breeding phenology, especially for single-brooded passerines. Hence, understanding the various selection pressures that influence how breeding phenology affects fecundity will ultimately help us to understand the diversity of avian life history strategies. Future studies should explicitly examine the relationship between breeding date and risk of nest predation, and design studies that build upon our results to better understand the underlying cause of these seasonal patterns.
